# Frontier impact of microRNAs in skeletal muscle research: a future perspective

**DOI:** 10.3389/fphys.2014.00495

**Published:** 2015-01-05

**Authors:** Wataru Aoi

**Affiliations:** Laboratory of Health Science, Graduate School of Life and Environmental Sciences, Kyoto Prefectural UniversityKyoto, Japan

**Keywords:** microRNAs, skeletal muscle, exercise, aging and longetivity, exosomes

## Abstract

MicroRNAs (miRNAs) are non-coding RNAs that can regulate the expression of mRNAs and proteins by degrading mRNA molecules or by inhibiting their translation. It has been predicted that miRNAs regulate approximately 60% of protein-coding genes that could be involved in a wide range of biological processes. Research over the last 5 years suggests that miRNAs play important roles in skeletal muscle function and several miRNAs have been identified as modulators of myogenesis, muscle mass, and nutrient metabolism in physiological and pathological states. In addition, some miRNAs can be incorporated into intracellular vesicles, released into the circulation, transported to other cells, and possibly function in other organs in an endocrine manner. This phenomenon might explain the interactions between skeletal muscles and other organs. Thus, far, several muscle-secreted miRNAs have been identified and their involvement in muscle biology has been debated. Based on the recent understanding, this perspective article describes the potential valuable role of miRNAs in skeletal muscle function, delineates its limitations, and outlines its future perspectives.

## Introduction

In recent years, there has been a significant advancement in our understanding of the importance and role of non-coding RNAs in several biological processes. Several non-coding RNAs, besides transfer and ribosomal RNAs, are involved in the transcriptional and translational regulation of other RNAs; these are referred to as functional RNAs. Among non-coding RNAs, microRNAs (miRNAs), which are small non-coding RNAs (approximately 19–22 nucleotides in length), are diverse and regulate over 1500 target mRNAs (Kozomara and Griffiths-Jones, 2011) by perfectly or partially hybridizing to complementary binding sites located in the 3′ untranslated regions of the mRNAs and by inhibiting translation via mRNA cleavage or steric hindrance. miRNAs hold promise for presenting new findings in cellular and molecular biological systems and contribute to the biomedical field by acting as biomarkers for clinical diagnosis and as drug targets for the treatment of diseases. The role and regulation of miRNAs in skeletal muscle function have also been evaluated; however, multiple aspects remain to be elucidated. The identification of an association between miRNAs and skeletal muscle function after several years of research has been a turning point in skeletal muscle research. Here, I discuss the advancements in skeletal muscle miRNA-based research and provide a perspective on its potential future implications.

## MicroRNA in muscular physiology and pathology

**Figure 1:**
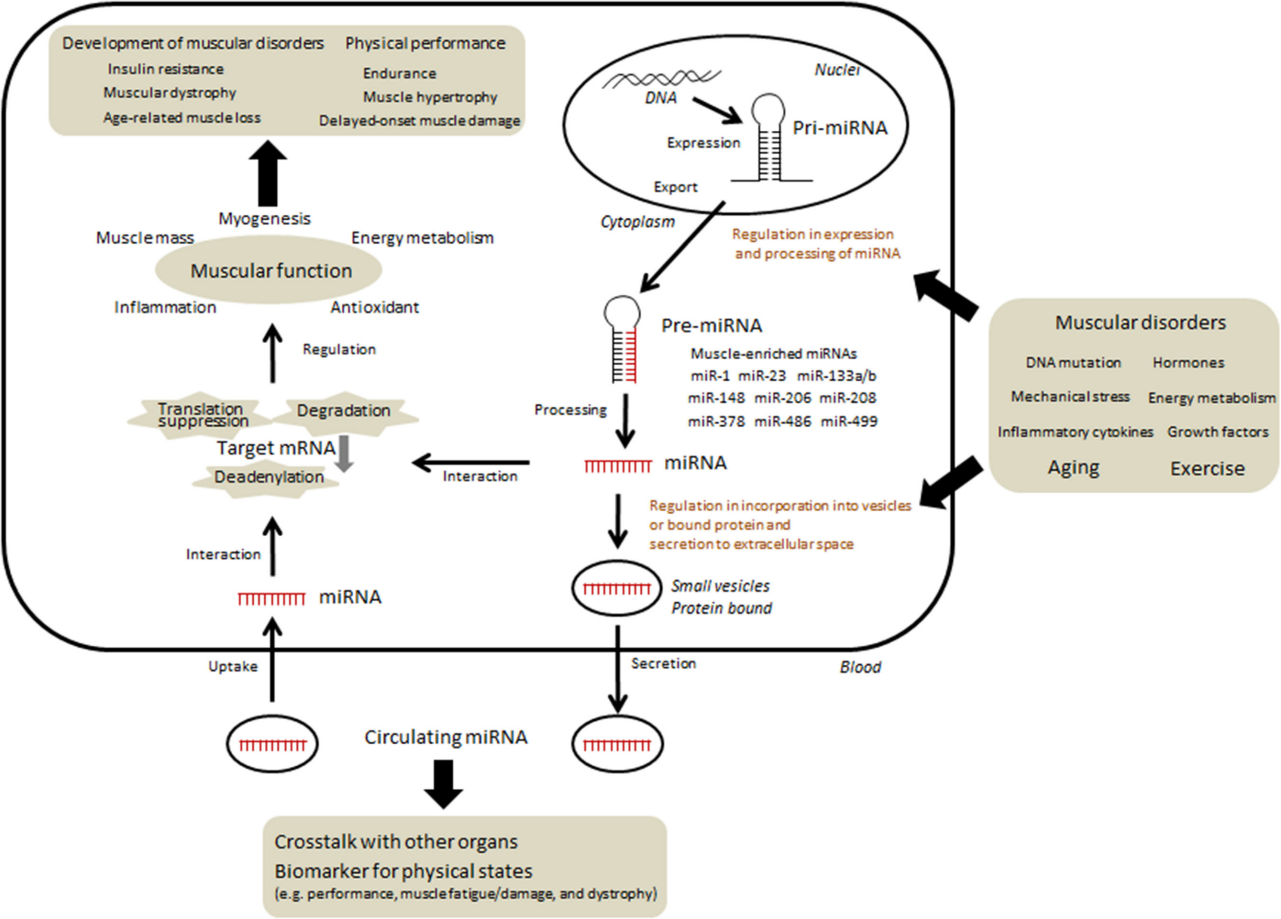
**Current understanding and hypothesis regarding the regulation and function of miRNAs in skeletal muscle**. The expression of several miRNAs changed according to various conditions such as physical activity, nutrients, diseases, and aging. In contrast, several miRNAs could function as modulators of myogenesis, muscle mass, and nutrient metabolism. Some of miRNAs can be taken up into intracellular vesicles and secreted into extracellular fluid. Circulating miRNAs migrate to skeletal muscle as well as other organs and may regulate certain functions. In addition, specific c-miRNAs may be useful in determining precise muscle-related events.

Research has suggested that miRNA-mediated gene regulation is a fundamental mechanism of post-transcriptional regulation and that it may have diverse functional effects. It has previously been shown that the levels of several proteins do not frequently correspond to the levels of their mRNA; this phenomenon could be partly attributed to translational regulation by miRNAs. For instance, the mRNA expression level of peroxisome proliferator-activated receptor gamma coactivator 1 alpha (PGC1-α) was markedly increased following exercise although the protein amount was not changed (Watt et al., 2004; Gibala et al., 2009), could be attributed to translational regulation by miRNAs. Indeed, earlier studies on muscular miRNA have shown that the levels of both miR-23 and miR-696, which hybridize with PGC1-α mRNA and reduce the protein amount, decrease in response to acute or chronic exercise (Safdar et al., 2009; Aoi et al., 2010). Approximately 60% of protein-coding genes may be regulated by miRNAs (Friedman et al., 2009). Therefore, miRNAs can influence the biological phenotype with regard to overall development, maintenance of homeostasis, cell death, carcinogenesis, and age-related changes by regulating protein content. Studies on skeletal muscles have suggested that several miRNAs could also function as modulators of myogenesis, muscle mass, and nutrient metabolism in skeletal muscle (Chen et al., 2006; Small et al., 2010; Dey et al., 2011; Gagan et al., 2011; Zhang et al., 2012; Hitachi et al., 2014), thus raising questions regarding the association of miRNAs with exercise-induced physiological changes, muscular pathogenesis, and age-related muscle dysfunction (Figure 1).

A tissue-specific miRNA is defined as a miRNA that is expressed in a specific tissue at levels that are >20-fold higher than its mean level in all other tissues (Lee et al., 2008). Several miRNAs are highly enriched in muscle tissue and are often referred to as myomiRs. Four myomiRs, namely, miR-1, miR-133a, miR-133b, and miR-206, together account for nearly 25% of miRNA expression in skeletal muscles in both humans and mice (Sempere et al., 2004; McCarthy, 2008). In addition, miR-208, miR-486, and miR-499 are encoded by muscle-specific genes such as *ankyrin* and *myosin heavy chain* (McCarthy et al., 2009; Small et al., 2010). The presence of miR-378 at high levels in muscle has been validated in several studies (Davidsen et al., 2011; Gagan et al., 2011). Therefore, skeletal muscle research is focused on the regulation of miRNAs and their association with muscle functions. A growing amount of evidences has suggested that these muscle-specific miRNAs, along with other miRNAs, affect various phenotypic changes in skeletal muscles, including exercise adaptation, immobilization, and muscular diseases (Eisenberg et al., 2007; Allen et al., 2009; Safdar et al., 2009; Aoi et al., 2010; Nielsen et al., 2010; Davidsen et al., 2011; Roberts et al., 2012; Russell et al., 2013; Alexander et al., 2014). In addition, expression of several miRNAs has been shown to be altered during aging in animals and humans (Hamrick et al., 2010; Mercken et al., 2013; Nielsen et al., 2014b; Rivas et al., 2014). Changes in the expression of some age-regulated miRNAs are reversed by calorie restriction, which is often adopted as a daily habit to prevent the development of age-related events and diseases (Mercken et al., 2013); this finding supports the significance of miRNA in aging. Recently, additional miRNAs that possibly play critical roles in muscle disorders related to diabetes, cancer, and inflammation have also been identified (Jiang et al., 2013; Chen et al., 2014; Georgantas et al., 2014; Rowlands et al., 2014; Sato et al., 2014). These findings provide novel information regarding the regulatory system of miRNAs, as described by several authors on this “research topics” (Zacharewicz et al., 2013; Aoi and Sakuma, 2014; Hitachi and Tsuchida, 2014; Sharma et al., 2014).

Another important characteristic of miRNAs is that a single miRNA regulates the expression of approximately hundreds of mRNAs and proteins by degrading mRNA molecules or by inhibiting their translation (Bartel, 2004; Djuranovic et al., 2012; Pasquinelli, 2012). This feature suggests that some miRNA's are functionally redundant and that the loss of functional regulation of a single miRNA does not always result in alterations in the expression of its target protein. Indeed, it has been observed that certain phenotypes are preserved despite impairments in specific regulatory miRNAs (Jin et al., 2009; Concepcion et al., 2012; Heyer et al., 2012), which is indicative of a cross-talk within complicated network of miRNAs involved in the modulation of skeletal muscle function. Thus, various miRNAs complement and cooperate with each other, making them essential molecular systems that maintain cellular homeostasis.

## Circulating microRNA and skeletal muscle

Several miRNAs are secreted from cells into the circulation or are taken up from circulation into cells, suggesting that minimal miRNA degradation occurs due to RNases present in body fluids (Mitchell et al., 2008). This may be attributed to the protection of miRNAs from RNases by intracellular small vesicles such as exosomes, microvesicles, and apoptotic bodies, or by their binding with non-vesicle-associated proteins such as lipoprotein particles (Vickers et al., 2011; Raposo and Stoorvogel, 2013). Modulation of the function of recipient cells by circulating miRNAs (c-miRNAs) could explain the communication between skeletal muscles and other organs in physiological and pathological conditions (Figure 1). It has been suggested that exercise transiently or adaptively changes the level of c-miRNAs in animals and humans (Baggish et al., 2011; Aoi et al., 2013; Bye et al., 2013; Sawada et al., 2013; Nielsen et al., 2014a), leading to post-transcriptional regulation of proteins associated with energy metabolism and angiogenesis in adipocytes, hepatocytes, and endothelial cells. The circulating levels of several muscle-enriched miRNAs are also altered in muscle disorders (Miyachi et al., 2010; Mizuno et al., 2011; Roberts et al., 2013) and may be involved in such pathologies. In addition, such c-miRNAs have a potential role as useful biomarkers owing to their stability in body fluids, which could determine the various interactions between tissues and reflect their physiological and pathological states. c-miRNAs have been evaluated as biomarkers for clinical diagnosis, particularly in cancer studies. As previously mentioned, since several specific miRNAs are associated with skeletal muscles, determining their c-miRNA levels may be useful for the estimation of muscle-related events.

It has previously been reported that several muscle-specific miRNAs can be detected in plasma and serum; the levels of these miRNAs are altered in some muscle disorders. Serum levels of several muscle-enriched miRNAs such as miR-1, miR-133, and miR-206 are elevated in Duchenne muscular dystrophy in humans and animals (Cacchiarelli et al., 2011; Mizuno et al., 2011; Hu et al., 2014). In addition to these miRNAs, other candidate c-miRNAs, such as miR-378 and miR-31 have been suggested to be associated with dystrophy in a recent human study (Vignier et al., 2013). Furthermore, circulating levels of muscle-enriched miRNAs such as miR-1, miR-206, and miR-499 are higher in patients with chronic obstructive pulmonary disease, who often exhibit reduced muscle fiber size and proportions compared with those of normal healthy subjects (Donaldson et al., 2013). Roberts et al. (2013) showed that the dystrophy-associated miRNAs dystromiRs exist in a protein-bound form but not in the extracellular vesicles. Levels of muscle-specific miRNAs (miR-1, miR-133a, miR-133b, and miR-206) are also higher in the serum of patients with rhabdomyosarcoma tumors than in the serum of control subjects (Miyachi et al., 2010). In muscular atrophy, the levels of miR-23a are decreased and miR-23a is secreted into the extracellular space after being taken up by exosomes (Hudson et al., 2014). In contrast, the majority of the miRNAs that are highly expressed in skeletal muscle are difficult to detect in circulation under normal physiological condition. Further, these miRNAs exhibit no changes in response to exercise (Baggish et al., 2011; Nielsen et al., 2014a), except for miR-486, the levels of which is decreased in response to acute and chronic moderate exercise associated with lower aerobic performance (Aoi et al., 2013). However, other researchers reported that the levels of several c-miRNAs are elevated after strenuous/prolonged exercise such as running a marathon and that, in particular, alterations in the levels of c-miR-1, c-miR-133a, c-miR-206, and c-miR-208 are closely associated with performance and muscle damage parameters (Baggish et al., 2014; Gomes et al., 2014; Mooren et al., 2014). These conflicting results could be attributed to variations in the kind, intensity, and duration of exercises evaluated in the different studies. It has been shown that levels of common miRNAs are influenced by both exercise and myopathy, rendering it difficult to establish the use of certain miRNAs as reliable biomarkers for muscle disease. This warrants the development of further parameters upon consideration of miRNAs as diagnostic tools.

## Limitations and prospects for future studies

The understanding of the role of microRNA in the field of skeletal muscle research has developed rapidly by examining muscle tissue and blood samples obtained not only from animal models but also from healthy human volunteers and patients. However, despite the rapid progress, several aspects regarding miRNAs are still unclear. Typical technical issues like the small size (length of approximately 20 nucleotides) and the sequence similarity between different miRNAs make it difficult to accurately quantify the levels of miRNAs and design specific probes and primers. miRNAs can exert their function even if they do not completely hybridize with their targets (Bartel, 2009; Fabian et al., 2010), which may be one of the reasons why several miRNAs show changes in expression that are common among exercised, dystrophied, and aging muscles. In addition, the development of protocols for quantification of c-miRNA in circulation is underway. It is important to avoid hemolysis during sampling not only because it is difficult to extract miRNAs from serum or plasma, but also because blood cells contain various miRNAs and can significantly affect the c-miRNA level (Pritchard et al., 2012). Strenuous exercise frequently induces hemolysis by mechanical, osmotic, and oxidative stress (Telford et al., 2003; Sentürk et al., 2005; Peeling et al., 2009), which could affect the profile of c-miRNAs.

Although a limited number of experimental systems have evaluated c-miRNAs (Kosaka et al., 2010; Mittelbrunn et al., 2011; Vickers et al., 2011; Hudson et al., 2014), mechanisms underlying the uptake of c-miRNAs into certain cells, their secretion, and their release into the circulation are still unclear. c-miRNA-binding proteins in exosomes and other extracellular vesicles need to be studied further. It should also be considered that miRNAs can bind to lipoproteins in circulation, in case of physiological and pathological conditions that affect the level of lipoproteins which could easily change with exercise and age. Furthermore, an approach involving the identification of miRNAs from other body fluids such as saliva, urine, and sweat to establish them as biomarkers is necessary for relatively simpler diagnosis, without the need for muscle biopsy or blood collection in clinical setting and in the field of athletics. Further research involving the characterization of detailed mechanisms and the physiological and pathological changes in miRNAs based on appropriate evaluation/assessment protocols is warranted. Nevertheless, the field of miRNA research is attractive and is expected to present more novel findings for researchers.

### Conflict of interest statement

The author declares that the research was conducted in the absence of any commercial or financial relationships that could be construed as a potential conflict of interest.
